# Authors' Reply

**DOI:** 10.1371/journal.pmed.0020186

**Published:** 2005-06-28

**Authors:** Alexander M Capron, Andreas Reis

**Affiliations:** World Health OrganizationGenevaSwitzerland

In response to our commentary [1] on their paper, “Designing Equitable Antiretroviral Allocation Strategies in Resource-Constrained Countries” [2], Wilson and Blower assert that we misunderstood both their analysis and the importance of their results [3]. Rather than “setting the record straight,” what may be needed is more effort to bridge the differences in disciplinary approach that create a greater appearance of disagreement than is actually the case.

On a factual level, we believe Wilson and Blower's results were appropriately described for the purposes of our commentary. As their letter points out, they applied the operations research methods to model the allocation of antiretrovirals (ARVs) among 17 health-care centers in KwaZulu–Natal, based on a hypothetical distribution of HIV/AIDS among the communities in that province. Their article characterizes this as “an elegant and simple theoretical framework,” but they object to our concluding that it could “inform policy-makers' decisions regarding the location of HIV services,” since they took the treatment sites as given. Yet their article compared the alternatives of using all 54 centers in the province, at one extreme, and of using only a single treatment site (in Durban), at the other extreme; in each case, the possibility of allocating to a larger number of centers is equivalent to the creation of additional centers closer to remote groups of patients.

Wilson and Blower write that geographic accessibility is improved if the number of health-care facilities is increased, and they calculated that it would be optimal if all 54 facilities in the province of KwaZulu–Natal distributed the medicines, instead of just 17. We took this result to confirm the need to reach out and build capacity. We are sorry if we were mistaken in assuming that Wilson and Blower would want to see their stated objective of ensuring fair distribution applied in the real-world context of many poor countries with a high HIV burden and where fairness in ARV care cannot be achieved solely by allocating resources among the existing sites.

A wider gap in perception can be seen in Wilson and Blower's repeated conflation of “optimal,” “equal,” and “equitable,” combined with their suggestion that decision makers who fail to apply their model must be following an “ad hoc approach.” The central point of our commentary was that various ethical theories reach very different conclusions about what result would be optimal, and that even among those aiming to achieve the greatest equity (rather than some other optimum), many would not take equality as the measure of equity. Wilson and Blower themselves recognize that apparent equality of access (in terms of distance to treatment) needs further study to determine whether patients can in fact access treatment. We need to know whether some distances are simply too far for patients to travel for chronic care, and when distances of equal length affect access very differently because of the characteristics of particular patient populations, transportation systems, and so forth.

Wilson and Blower seem unwilling to accept the notion that, in the furtherance of a rational strategy to achieve equity, some health authorities might decide, for example, to allocate a disproportionate share of ARVs to traditionally disadvantaged populations. Wilson and Blower's model could still be useful in allocating resources among the centers chosen (or established) to reach the target population, but the calculation would have to take account of more information about the centers and the population, lest assumptions about catchment areas produce a formal equality that does not translate into actual equality in access, much less into equitable access in light of all relevant factors.

Plainly, we share Wilson and Blower's aim of optimizing countries' responses to the tragedy of treatable, but untreated, HIV/AIDS. Any tools that are useful to that end are welcome. But besides using models to distribute ARVs in a way that optimizes spatial equality, governments that want to achieve equity will need also to overcome nongeographic barriers to accessing treatment. These include ignorance, stigma, discrimination, and outright criminalization of vulnerable groups, as well as fees at point of service that are prohibitive for the poor. All of these are given attention within the context of the “3 by 5” program of the World Health Organization and the United Nations Joint Programme on HIV/AIDS, including in the guidance document on equitable access to ARV treatment cited in our commentary [4].
